# Sorption of Water Vapor in Poly(L-Lactic Acid): A *Time-Resolved* FTIR Spectroscopy Investigation

**DOI:** 10.3389/fchem.2019.00275

**Published:** 2019-04-24

**Authors:** Marianna Pannico, Pietro La Manna

**Affiliations:** Institute of Polymers, Composites and Biomaterials, National Research Council of Italy, Pozzuoli, Italy

**Keywords:** water vapor, diffusion, FTIR spectroscopy, PLLA, molecular interaction

## Abstract

In this contribution the sorption of water vapor in Poly(L-lactic acid) (PLLA) was studied by time-resolved FTIR spectroscopy. The collected FTIR data were analyzed by complementary approaches such as difference spectroscopy, two-dimensional correlation spectroscopy (2D-COS), and least-squares curve-fitting analysis which provided information about the overall diffusivity, the nature of the molecular interactions among the polymer and the penetrant and the dynamics of the various molecular species. The diffusion coefficient were evaluated as a function of vapor activity and were found in good agreement with previously reported values. The system showed a Fickian behavior with diffusivity increasing with penetrant concentration. Two distinct water species (first-shell and second-shell layers) were detected and quantified by coupling FTIR and gravimetric measurements.

## Introduction

Over the past decade, the use of polymeric biodegradable materials has increased substantially because of their versatility in a variety of applications as well as for the increasing environmental concern. They are widely used in the pharmaceutical, medical, and packaging fields due to their unique properties such as biocompatibility, biodegradability, eco-friendliness, and processability (Ikada and Tsuji, 2000; Temenoff and Mikos, 2000; Chen et al., 2002; Noda and Ozaki, 2004; Nair and Laurencin, 2007; Siracusa et al., 2008; Sabir et al., 2009; Armentano et al., 2010; Leja and Lewandowicz, 2010). Among the wide family of biopolymers, Poly(lactic acid) (PLA), and, in particular, its L-isomer, PLLA, has gained prominence owing to its excellent biocompatibility and good mechanical properties. Commercially, PLLA has been introduced in food packaging applications including oriented and flexible films, extruded and/or thermoformed packages for food and beverage containers, cups, overwrap, and blister packages (Tullo, 2000; Auras et al., 2004; Sagar et al., 2007; Tawakkal et al., 2014). In the realm of biomaterials, Dürselen et al. (2001) demonstrated that PLLA fibers are ideally suited for ligament and tendon reconstruction as well as stents for vascular and urological surgery. PLLA based microspheres were also used as injectable material in facial reconstructive surgery and in drug delivery systems (DDS) for the administration of a wide variety of medical agents (Imola and Schramm, 2002; Eppley et al., 2004; Tyler et al., 2016). PLLA contains flexible ester bonds whose hydrolytic degradation is caused by water diffusing into the bulk material. The hydrolytic products are non-harmful and non-toxic monomers/oligomers because they are metabolized via the citric acid (Krebs) cycle. For both medical and packaging applications, hydrolysis would be one of the most important degradation mechanism to account for. The PLLA hydrolysis behavior in biomedical devices such as implants and carriers in DDS, has been widely investigated in different media at various temperatures (Göpferich, 1996; Li, 1999; Liu et al., 2000; Tsuji et al., 2000, 2003, 2011; Tsuji and Miyauchi, 2001; Fukuda et al., 2002; Kikkawa et al., 2002; Yuan et al., 2002). Literature data are mainly concerned with the effects of water in the liquid state. Only few studies have been reported on the PLLA hydrolysis carried out by water vapor (Ho et al., 1999; Copinet et al., 2004; Holm et al., 2006). Water sorption in PLA significantly affects the physico-chemical properties of the polymer matrix. Rocca-Smith et al. (2017) clearly showed that the stability of PLA was also influenced by the water physical state. It is evident that, in all the above applications, the diffusion of water in the bulk material represent one of the major issue to be considered. In this contribution, a molecular level description of water vapor diffusion in a fully amorphous PLLA matrix is reported. Among the different spectroscopic techniques employed for the investigation of water–polymer systems (Rothwell et al., 1984; Taylor et al., 2001) (Solid-state NMR, Raman, neutron scattering, light scattering) FTIR spectroscopy has been demonstrated to be very powerful because of its sensitivity toward H-bonding detection and its sampling flexibility (Cotugno et al., 2001; Scherillo et al., 2013).

We report on a *time-resolved* FTIR study performed at different relative pressures of water vapor. The spectral data have been analyzed by different and complementary approaches, namely, difference spectroscopy (DS), least-squares curve fitting (LSCF), and 2-D correlation spectroscopy (2DCOS) which allowed us to isolate the spectrum of the penetrant and to improve the resolution of its complex band profile in the ν(OH) frequency range. The above techniques, taken together, provided information on the nature, number, and dynamic behavior of the water species present in the investigated system. By coupling the spectroscopic data with gravimetric measurements, carried out in the same conditions of temperature and vapor pressure, we were able to quantify the water species population.

## Experimental

### Materials

PLLA was a commercial grade product (Ingeo Biopolymer 2003D) kindly provided by Nature Works (Minnetonka, MN, USA). It had ${\bar{M}}_{n}=\left(79.4+1.1\right)\;kDa,\;{\bar{M}}_{w}=\left(121.3+0.5\right)\;kDa$ and a polydispersity of 1.53 ± 0.02. The present PLLA resin, in the form of pellets, contains, according to the supplier, 4% D-lactic acid isomer; it has a density of 1.240 g/cm^3^, a melting temperature of 145–170°C, a glass transition temperature (T_g_) of 55–58°C, and a crystallinity (maximum attainable) of 35%. Chloroform, 99.8% purity, was purchased from Sigma-Aldrich (Milan, Italy) and used with no further purification.

### Film Preparation

A 20 wt% solution of PLLA in chloroform was spread onto a glass plate with a Gardner knife to obtain a film thickness of 46 ± 5 μm. The PLLA film was kept overnight at room temperature to remove most of the solvent. Final drying was accomplished in a vacuum oven at 40°C for 10 days. No residual solvent was detected by spectroscopic (FTIR) and gravimetric (TGA) measurement.

### FTIR Sorption Measurements

*Time-resolved* spectra were collected in transmission mode during sorption/desorption cycles of water vapor in the samples. The experiments were performed using a vacuum-tight FTIR sorption cell in which a free standing PLLA film is exposed from both sides to water vapor at constant temperature (35°C) and selectable relative pressures (*p/p*_0_ = 0–0.5) of the penetrant. The sorption cell was accommodated in the sample compartment of a suitably modified FTIR spectrometer [Spectrum 100 from Perkin-Elmer (Norwalk, CT)], equipped with a Ge/KBr beam splitter and a wide-band DTGS detector. The cell was directly connected through service lines to a water reservoir, a turbo-molecular vacuum pump, a pressure transducer [MKS Baratron 121 (Andover, MA); full scale, 100 Torr; resolution, 0.01 Torr; accuracy, ± 0.5% of the reading] and a Pirani vacuometer. Full details of the experimental apparatus are reported in Cotugno et al. (2001). Before each sorption measurement, the sample was dried under vacuum, overnight, at 35°C in the same apparatus used for the test. The instrumental parameters for data collection were set as follows: resolution = 2 cm^−1^; optical path difference (OPD) velocity = 0.2 cm/s; spectral range, 4,000–600 cm^−1^. Spectra were acquired in the single-beam mode for subsequent data processing. Automated data acquisition was controlled by a dedicated software package for *time-resolved* spectroscopy (Timebase, from Perkin-Elmer).

### Gravimetric Measurements

For calibration purposes, gravimetric measurements were performed using a microbalance Q5000 SA apparatus (produced by TA Instruments, New Castle, DE, USA) that is a fully automated gravimetric water vapor sorption analyzer, operating in the 5–85°C temperature range. The sample was exposed to a series of humidity step changes at a constant temperature of 35°C. The relative humidity in the sample chamber is dynamically controlled in the 0–98% RH range, with an accuracy of ±1% RH, by mixing, in due proportion, a dry and a gaseous nitrogen stream saturated with water vapor by means of electronic mass flow controllers. Integral sorption runs were performed at four selected values of relative pressure, *p/p*_0_, i.e., 0.202, 0.396, 0.582, and 0.764. Prior to each sorption test, the sample was dried in the microbalance under dry nitrogen steam until a constant weight was attained. Further details on the experimental apparatus and data treatment are reported in Scherillo et al. (2012b).

### FTIR Data Analysis

Full absorbance spectra (i.e., sample plus absorbed water) were obtained using a background collected on the empty cell at the same relative pressure of water vapor used for the sorption measurement. The spectra representative of absorbed water were obtained by using the difference spectroscopy (DS) technique, i.e., by subtracting the spectrum of the dry sample from that of the sample equilibrated at different *p/p*_0_ values:

(1) $$\begin{array}{l} A_{d}\left(\nu \right)=A_{s}\left(\nu \right)-k\cdot A_{r}\left(\nu \right) \end{array}$$

where *A(*ν*)* is the absorbance at frequency ν and the subscripts *d, s*, and *r* denote, respectively, the difference spectrum, the sample spectrum (*wet* specimen), and the reference spectrum (*dry* specimen). *k* is an adjustable parameter which allows to compensate for thickness differences (if any) between the sample and the reference spectra. It was experimentally verified that negligible thickness changes take place during sorption; therefore, the *k* values were consistently taken as unity. The DS procedure allowed us to eliminate the interference of the polymer spectrum in the analytical ranges of interest [3,800–3,400 cm^−1^, ν(OH), and 1,660–1,550 cm^−1^, δ(HOH)]. Separation of multicomponent bands into individual peaks was achieved by a least-squares curve fitting (LSCF) algorithm based on the Levenberg–Marquardt method (Marquardt, 1963). The peak function was a mixed Gauss–Lorentz line-shape of the form:

(2) $$\begin{array}{l} f\left(x\right)=\left(1-Lr\right)H\exp-\left[{\left(\frac{x-x_{0}}{FWHH}\right)}^{2}\left(4\ln\;2\right)\right]+Lr\frac{H}{4{\left(\frac{x-x_{0}}{FWHH}\right)}^{2}+1} \end{array}$$

where *x*_0_ is the peak position; *H* the peak height; *FWHH* the full-width at half height and *Lr* is the fraction of Lorentz character. In order to keep the number of adjustable parameters to a minimum, the baseline, the number of components and the band-shape (*Lr* parameter) were fixed, allowing the curve-fitting algorithm to optimize *FWHH, H*, and *x*_0_ for the individual components.

### Two-Dimensional Correlation Spectroscopy (2D-COS) Analysis

The experimental spectra for 2D-COS analysis were pre-processed to avoid the occurrence of artifacts due to baseline instabilities and other non-selective effects. The frequency region of interest (3,900–3,300 cm^−1^) was isolated and offset to zero absorbance. Generalized 2D-IR analysis was performed by a script written in house with the MATLAB programming language (Mathworks, Natick, MA). The MATLAB environment also provided the tools for the graphical representation of the correlation spectra (contour plots, 3D images). The algorithm proposed by Noda relying on the Hilbert transform (Noda, 2000) was used for the numerical evaluation of the correlation intensities. The 2D correlation analysis was performed on an evenly spaced sequence of 100 spectra collected at a constant sampling interval of 0.98 s. The analyzed time-span (98 s) was sufficient to attain sorption equilibrium (vide infra). The notation adopted to identify the peaks appearing in the correlation spectra is that described in Musto et al. (2007).

## Results and Discussion FTIR Spectroscopy

**Figure 1:**
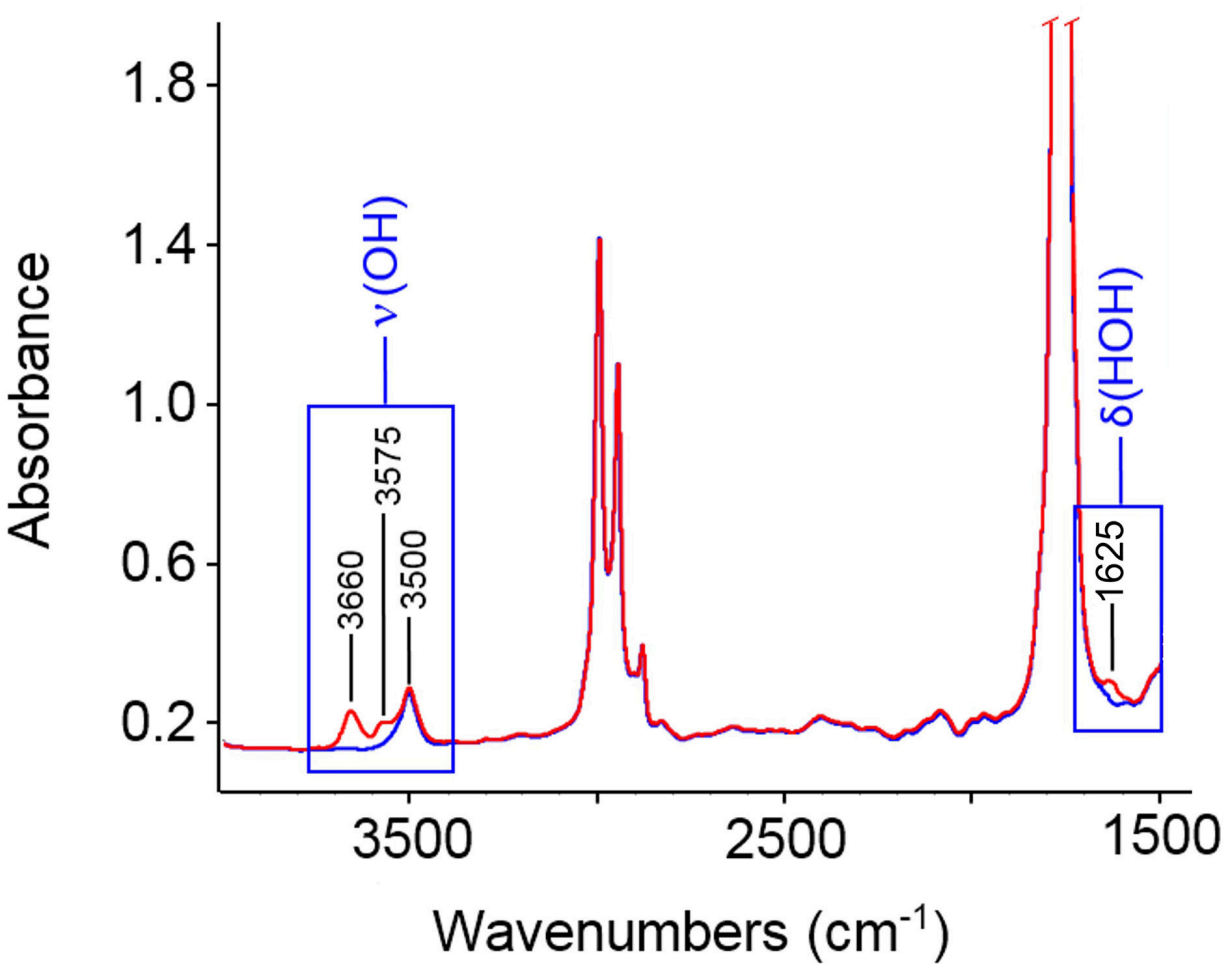
FTIR spectra of: fully dried PLLA film (blue trace); the same film equilibrated at *p/p*_0_ = 0.5 (red trace).

**Figure 2:**
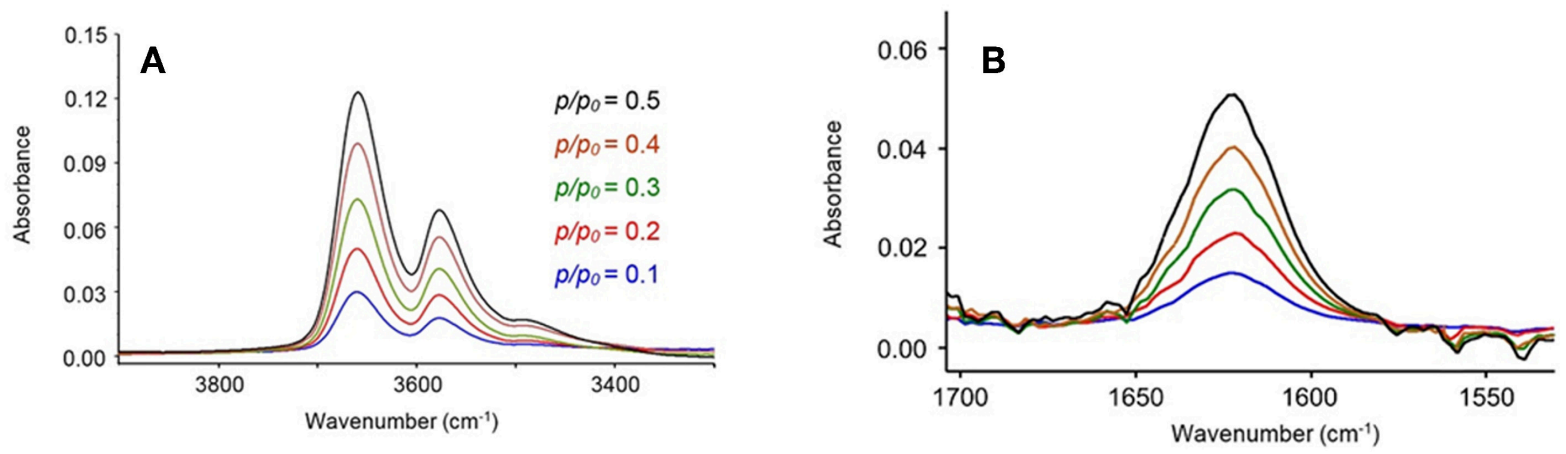
Difference spectra in the 3,900–3,300 cm^−1^ range **(A)** and in the 1,700–1,540 cm^−1^ range **(B)** for the PLLA film equilibrated at different relative pressures of water vapor.

In Figure 1 are reported the FTIR spectra of the fully dried PLLA film (blue trace) and of the same film equilibrated at a relative pressure of water vapor, *p/p*_0_, equal to 0.5 (red trace). Sorbed water displays characteristic bands around 3,660 cm^−1^ [ν(OH)] and 1,625 cm^−1^ [δ(HOH)]. Difference spectroscopy (DS) allows us to suppress the interference of the substrate and to isolate the spectrum of the penetrant. This is represented in Figures 2A,B in the 3,900–3,200 cm^−1^ range and in the 1,700–1,500 cm^−1^ range, respectively. The spectra were collected at the indicated relative pressures of water vapor. Both ν(OH) and δ(HOH) bands increase with relative pressure and the bandshapes are very reproducible, which suggests that, in the explored *p/p*_0_ range the molecular interactions formed between the probe and the polymer substrate (and, eventually, the probe self-association) do not depend on H_2_O concentration. The featureless and symmetrical profile of the δ(HOH) band reflects the very low sensitivity of the δ-mode to H-bonding (Murthy and Rao, 1968). This characteristic makes the bending peak suitable for analytical purposes. The stretching band is more complex, owing to the high sensitivity of these modes to the molecular environment.

Two well-defined maxima are observed at 3,660 and 3,578 cm^−1^, suggesting the presence of multiple water species involved in different types of H-bonding interactions. These results confirm that in-depth analysis of the ν(OH) profile will provide molecular level information on the system under scrutiny. Preliminary attempts at curve resolution of the traces in Figure 2A by LSCF analysis using only two components were unsuccessful, regardless of the adopted bandshape.

This suggests a more complex structure of the experimental profile, which had to be explored by resolution-enhancement approaches.

**Figure 3:**
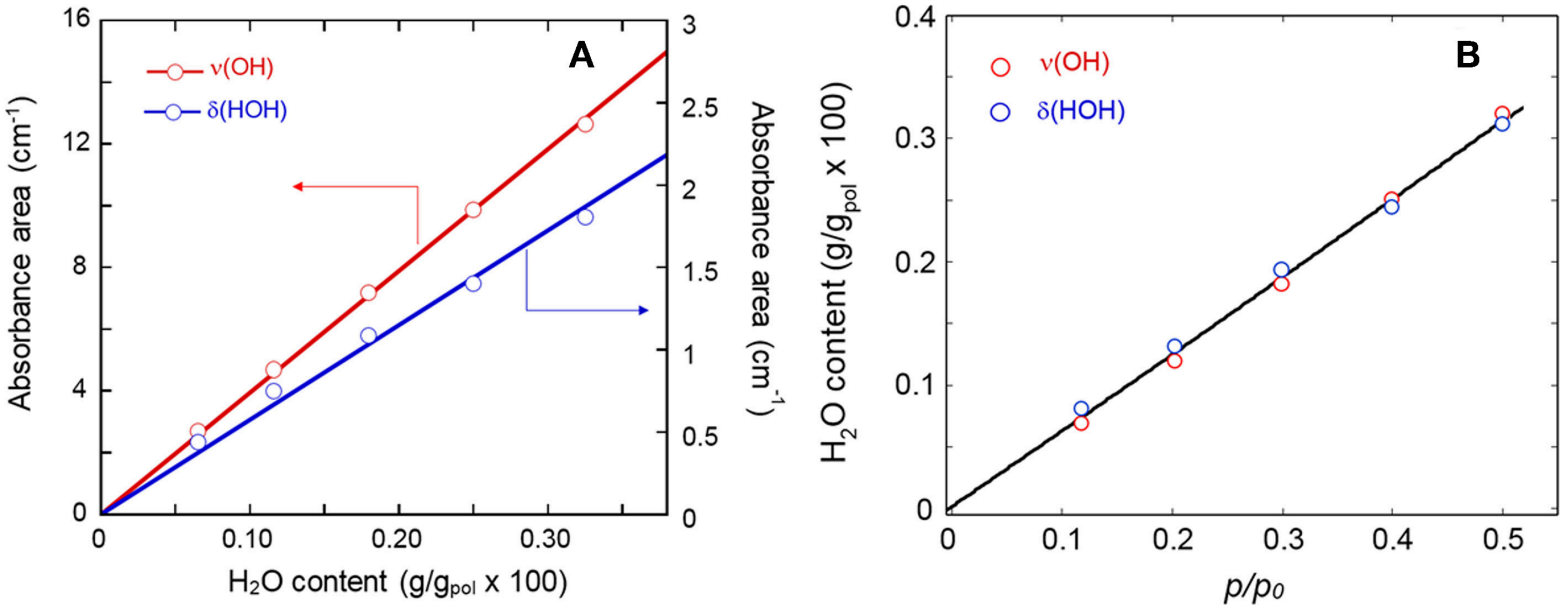
**(A)** Calibration curves (Absorbance vs. gravimetric concentration) for the ν(OH) and the δ(HOH) bands of sorbed H_2_O. **(B)** Sorption isotherm as evaluated from the two analytical signals.

In Figure 3A is represented the correlation between the absorbance area of the ν(OH) and the δ(HOH) bands with the amount of sorbed water measured gravimetrically. The linear trend through the origin confirms the validity of the Beer-Lambert relationship and allows us to use the photometric data for the quantitative monitoring of the diffusion kinetics and for measuring solubility vs. relative-pressure isotherms.

**Figure 4:**
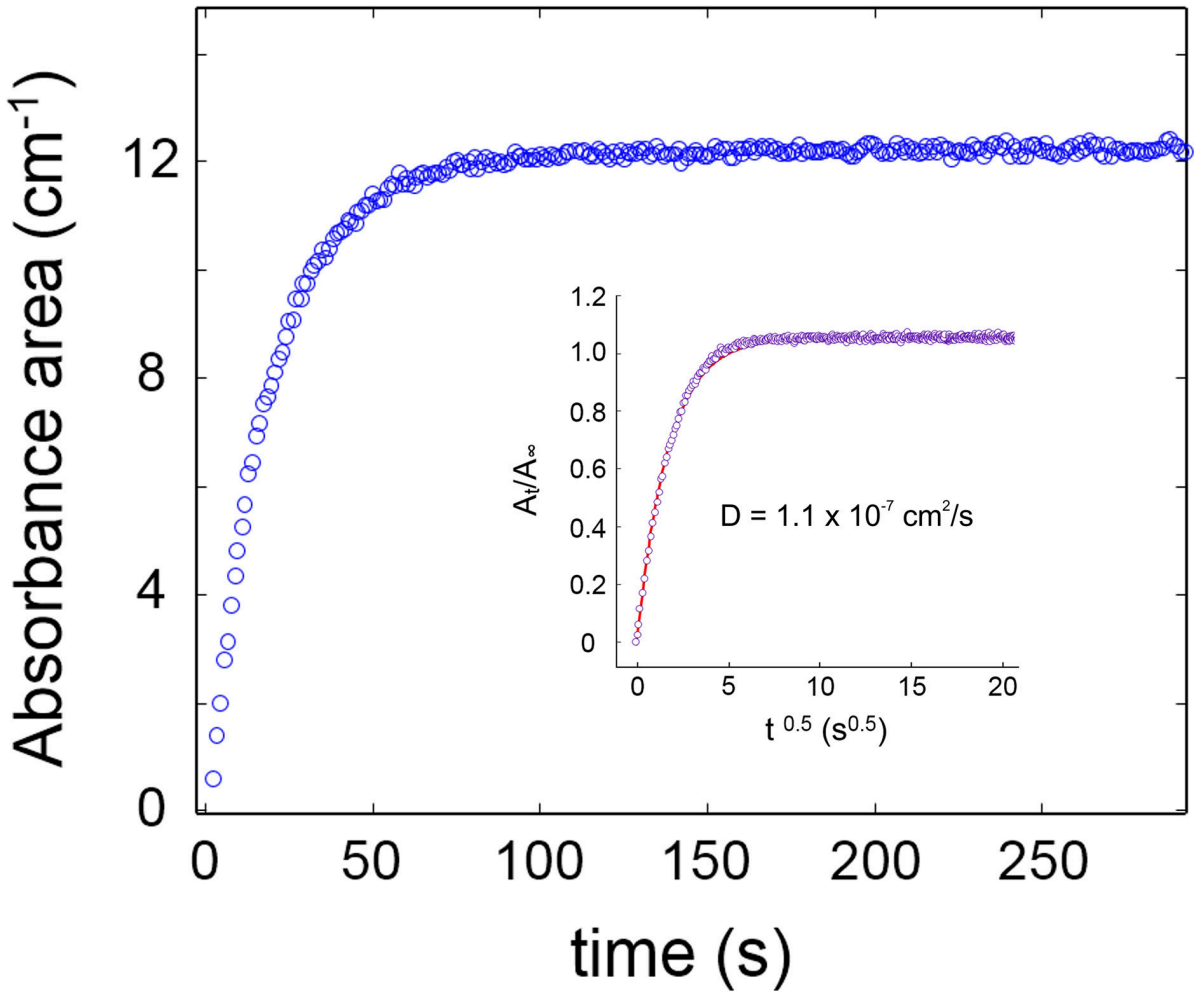
Absorbance of the ν(OH) band of water as a function of time for a sorption experiment at *p/p*_0_ = 0.5. The inset displays the Fick's diagram obtained therefrom. Open circles refer to experimental data points; the continuous line is the least-squares regression with model Equation (3).

The sorption isotherm at 35°C in the *p/p*_0_ range 0.1–0.5 is reported in Figure 3B, while Figure 4 shows the sorption kinetics at *p/p*_0_ = 0.5. As expected, data taken from the two analytical signals are essentially coincident; the isotherm is linear in the explored pressure range and the maximum mass uptake is 0.33 wt%. This value is low and only slightly higher than those for other semicrytalline polyesters of similar molecular structure [PCL, poly(propylene sebacate)], on account of the hydrophobic character of these polymers. More on this later. The kinetic behavior was modeled by the Fick's second law of diffusion expressed in terms of absorbance, which, for the case of a plane sheet exposed to an equal penetrant activity on both sides, can be written as (Crank, 1975):

(3) $$\begin{array}{l} \frac{A\left(t\right)}{A_{\infty }}=\frac{M\left(t\right)}{M_{\infty }}=1-\frac{8}{\pi^{2}}\overset{\infty }{\underset{m=0}{\sum }}\frac{1}{{\left(2m+1\right)}^{2}}\;exp\left[\frac{-D{\left(2m+1\right)}^{2}\pi^{2}t}{L^{2}}\right] \end{array}$$

In Equation (3) *A(t)* and *A*_∞_ represent the integrated absorbance of the ν(OH) band at time *t* and at equilibrium, *L* is the film thickness and *D* is the mutual diffusivity.

The model consistently simulates the experimental data in the whole time range (see inset of Figure 4), and the *A(t)/A*_∞_ vs. t^0.5^ curve is linear up to an ordinate value of 0.6, which demonstrates the Fickian behavior of the system. This result is in line with earlier literature reports on polyesters belonging to the same family (Musto et al., 2014; Scherillo et al., 2016).

**Figure 5:**
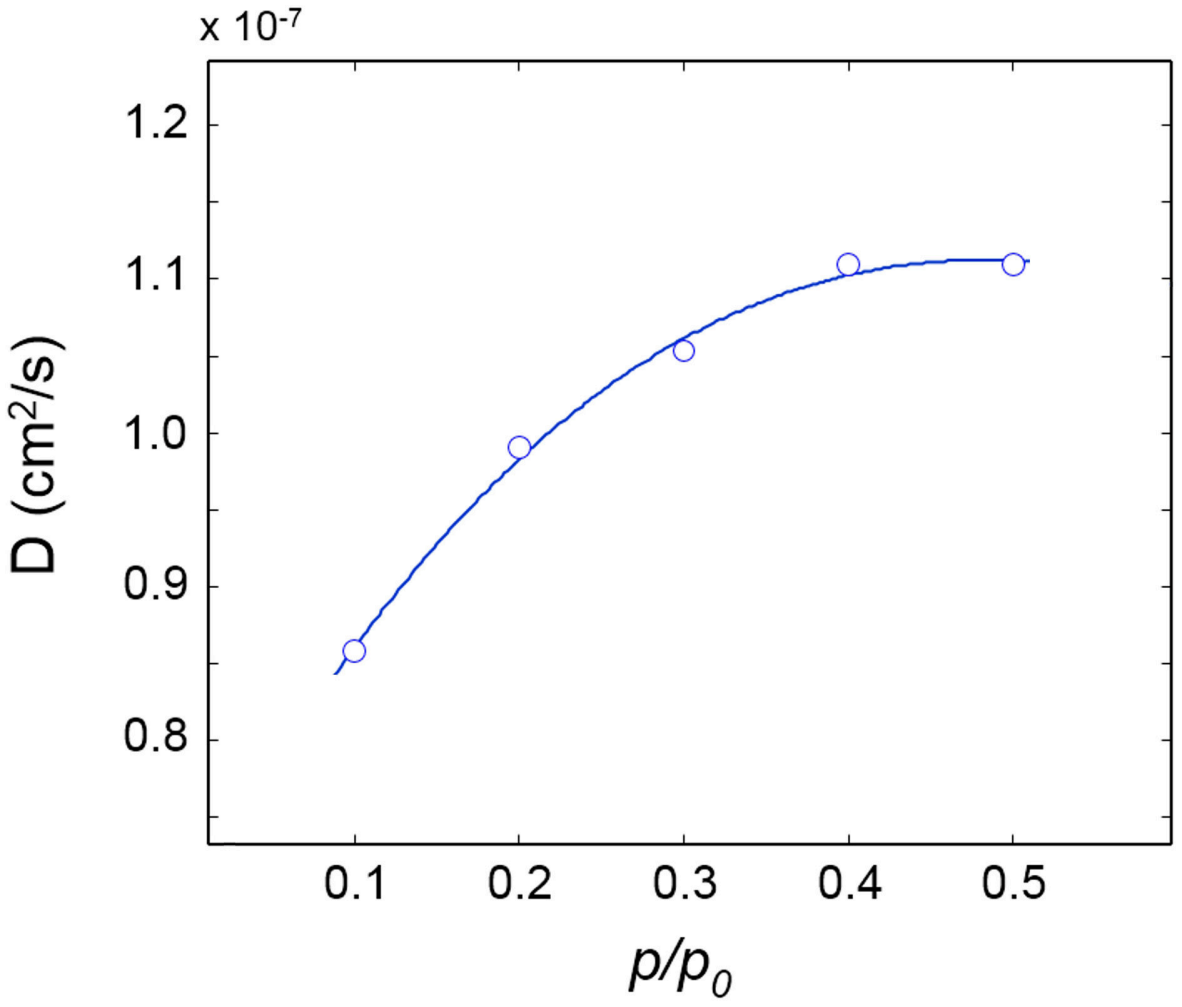
Diffusivity of H_2_O in PLLA as a function of relative pressure of water vapor. The connecting line is for eye guidance.

Sorption kinetics were monitored at five *p/p*_0_ values from 0.1 to 0.5 and the diffusion coefficients, *D*, evaluated therefrom are plotted as a function of *p/p*_0_ in Figure 5.

The *D*-value reported in the literature (0.7 × 10^−7^ cm^2^/s by gravimetry) (De Santis et al., 2015) is found in good agreement with the present spectroscopic determinations; an increasing trend of *D* with the water vapor pressure is also noted in Figure 5, which indicates that the mutual diffusivity coefficient is a growing function of the penetrant concentration.

This behavior is generally associated to a swelling effect of the penetrant, which increases the available free volume, with the consequence of enhancing the mobility of the diffusing molecules. In the present case, no direct spectroscopic evidence is found for the swelling of the sample upon sorption. However, a possibility exists that, while the swelling is so small to remain below the limits of detection, it is still sufficient to produce a sizeable effect at the molecular level. The present diffusivity data only allow us a tentative interpretation; a deeper analysis supported by thermodynamic and/or MD modeling is currently underway.

### 2D-COS Analysis

The 2D-COS technique was shown to be a powerful method for studying molecular interactions that produce broad, poorly resolved features (Noda and Ozaki, 2004; Galizia et al., 2014). It is a perturbative technique by which a system initially at equilibrium is subjected to an external stimulus: a correlation analysis is performed on the spectral response (absorbance, in the present case) as a function of a third common variable related to the perturbing function (time, in the present case).

**Figure 6:**
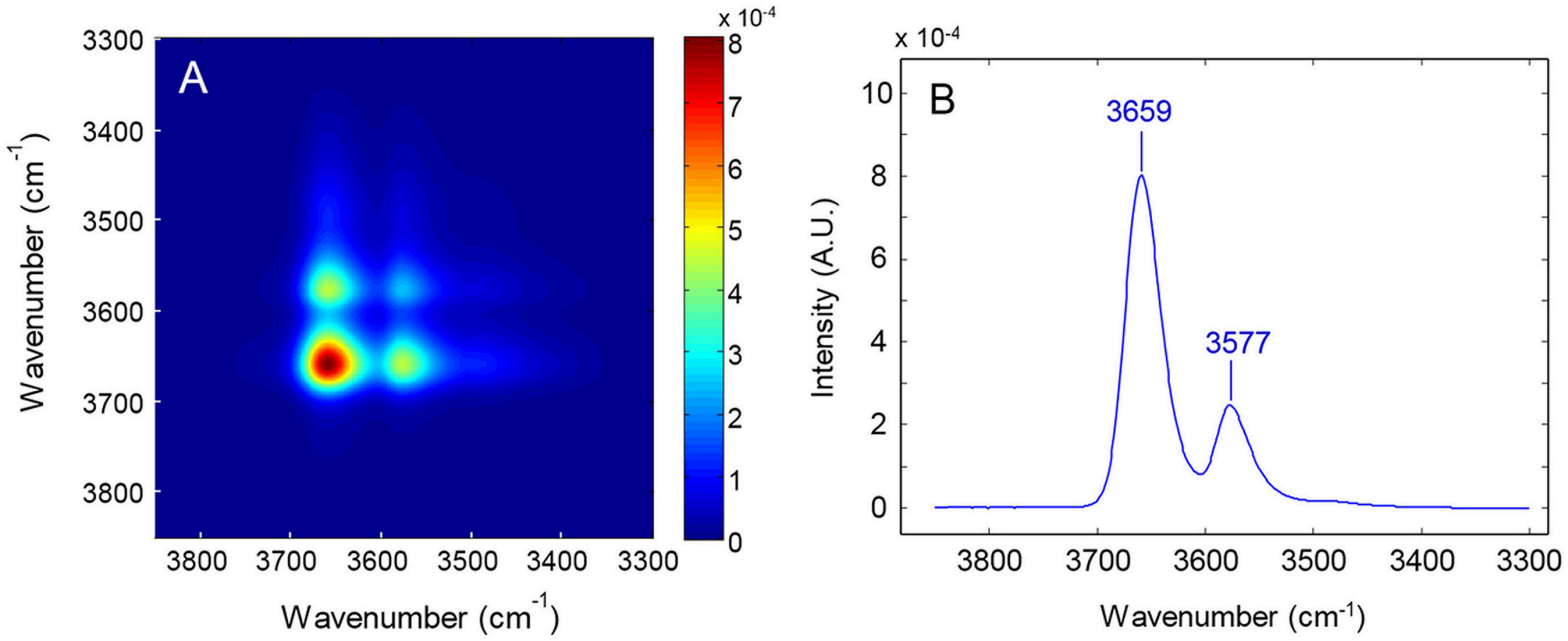
**(A)** 2D-COS synchronous color-map obtained from the *time-resolved* spectra collected during the sorption experiment at *p/p*_0_ = 0.5. **(B)** Power spectrum calculated therefrom.

In Figures 6A,B are represented, respectively, the synchronous spectrum in the 3,800–3,300 cm^−1^ range, obtained from the time-resolved spectra collected during the sorption experiment performed at *p/p*_0_ = 0.5 and the power spectrum, i.e., the autocorrelation profile taken across the main diagonal. The synchronous map displays the autopeaks (and the corresponding cross-peaks) already identified in the frequency profile; in the power-spectrum the two components are fully resolved and there is no evidence of further spectral features, in contrast with the results of the LSCF analysis.

**Figure 7:**
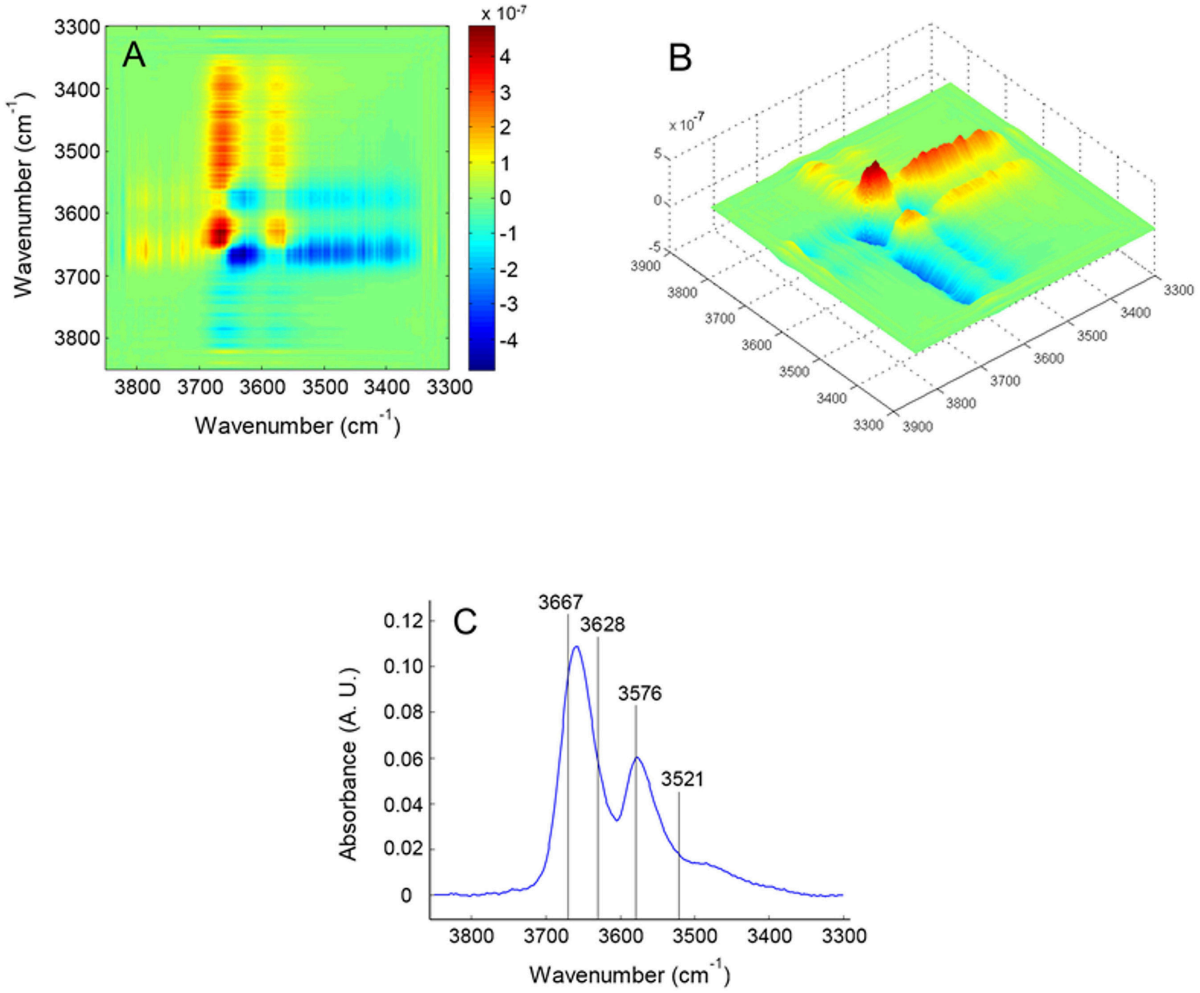
2D-COS asynchronous map obtained from the *time-resolved* spectra collected during the sorption experiment at *p/p0* = 0.5. The map is represented as an intensity–color map **(A)** and as a 3-D image **(B)**; **(C)** presents the frequency spectrum with the position of the components identified by 2D-COS analysis.

In Figure 7 is displayed the asynchronous spectrum in the form of a contour-map (Figure 7A) and as a 3D iso-intensity surface (Figure 7B) which highlights finer details on the shape of the correlation bands. The components identified in the asynchronous map are indicated in the frequency spectrum of Figure 7C. The results of the analysis are summarized in Table 1. The specificity and the resolution enhancement brought by asynchronous correlation allows us to identify two components in the main frequency peak at 3,659 cm^−1^, located, respectively, at 3,667 and 3,628 cm^−1^. These are readily recognized in the form of two well-developed cross-peaks (in the lower side of the map with respect to the main diagonal) at [3,628–3667 (–)], [3,576–3,628 (+)]. The shape of these two cross-peaks is typical of a correlation between two sharp signals. Two more correlation bands occur at [3,521–3,667 (–)] and [3,521–3,576 (–)]. These are less defined and display the elongated shape characteristic of a correlation between a sharp signal and a much broader band. In particular, the band at [3,521–3576 (–)] is only slightly above the noise level (see Figures 7A,B), reflecting the low intensity of the signal at 3,521 cm^−1^. The two above features clearly identify the presence of a broad component approximately centered at 3,521 cm^−1^, already suggested by the preliminary LSCF analysis. The asynchronous map displays a more detailed pattern and is richer of information than the synchronous.

**Table 1:** Position, sign, and type of the correlation peaks appearing in the synchronous and asynchronous spectra.

***ν_1_* (cm^−1^)**	***ν_2_* (cm^−1^)**	**Sign^a^**	**Type^b^**	**Trend^c^**
**SYNCHRONOUS**				
3,659	3,659	+	A	>>
3,577	3,577	+	A	>>
3,659	3,577	+	C	>>
***ν_1_* (cm^−1^)**	***ν_2_* (cm^−1^)**	**Sign**^**a**^	**Type**^**b**^	**Rate of change**
**ASYNCHRONOUS**				
3,628	3,667	–	C	3,628 < 3,667
3,576	3,628	+	C	3,576 > 3,628
3,521	3,667	–	C	3,521 < 3,667
3,521	3,576	–	C	3,521 < 3,576

a *The sign refers to the cross-peaks in the lower side of the spectrum, with respect to the main diagonal. The sign of the cross-peaks in the upper side can be deduced on the basis of the symmetry properties of the respective matrices (synchronous, symmetric; asynchronous, antisymmetric)*.

b *A, autopeak (along the main diagonal); C, cross-peak (off-diagonal)*.

c *>, increasing peak; <, decreasing peak*.

This effect is related to the fact that in the former case resolution enhancement occurs via two distinct mechanisms, i.e., the spreading of the spectral data over a second frequency axis, and the vanishing of the asynchronous correlation intensity for signals evolving at the same rate. A detailed discussion of the latter effect is reported in Musto et al. (2018). In the synchronous spectrum only the first mechanism is operative.

To summarize, 2D-COS identified four components in the ν(OH) range: three are sharp and are located at 3,667, 3,628, and 3,576 cm^−1^, the fourth is much broader and is so weak to be barely detectable in the frequency spectrum. Taking into account the correlation relationships from the 2D-COS maps, the following interpretation can be advanced: the four components are arranged pair-wise, the two signals centered at 3,667 and 3,576 cm^−1^ evolve synchronously and at a different rate with respect to the couple at 3,628–3,521 cm^−1^ that is also synchronously correlated. Applying the Noda correlation rules (Noda and Ozaki, 2004) the sign of the cross-peaks reveals that, in the sorption experiment the doublet at 3,655–3,562 cm^−1^ grows faster than the doublet at 3,611–3,486 cm^−1^.

The above findings can be interpreted considering that a single water molecule produces two OH-stretching modes (in-phase at lower frequency and out-of-phase at higher frequency). Thus, the two couples of signals suggest the presence of two distinct water species. The doublet at 3,667–3,576 cm^−1^ is assigned, respectively, to the ν_as_ and the ν_s_ modes of isolated water molecules interacting with the PLLA carbonyls, while the second couple at 3,628–3,521 cm^−1^ originates from a self-associated water species. It has been demonstrated that, when aggregates of the type C = O….H–O–H (Iwamoto et al., 2003) are formed, the “free” O–H bonds in the complex produces a characteristic signature at 3,690 cm^−1^. The absence of this feature, coupled with the 2D-COS results which detected only two H_2_O species, provides support for the conclusion that in the present system the amount of H_2_O molecules forming an H-bonding interaction with a single carbonyl group is negligible. The stoichiometry of the carbonyl-to-water interaction is thus 2:1, i.e., of the type:

–C = O….H–O–H….O = C–.

**Figure 8:**
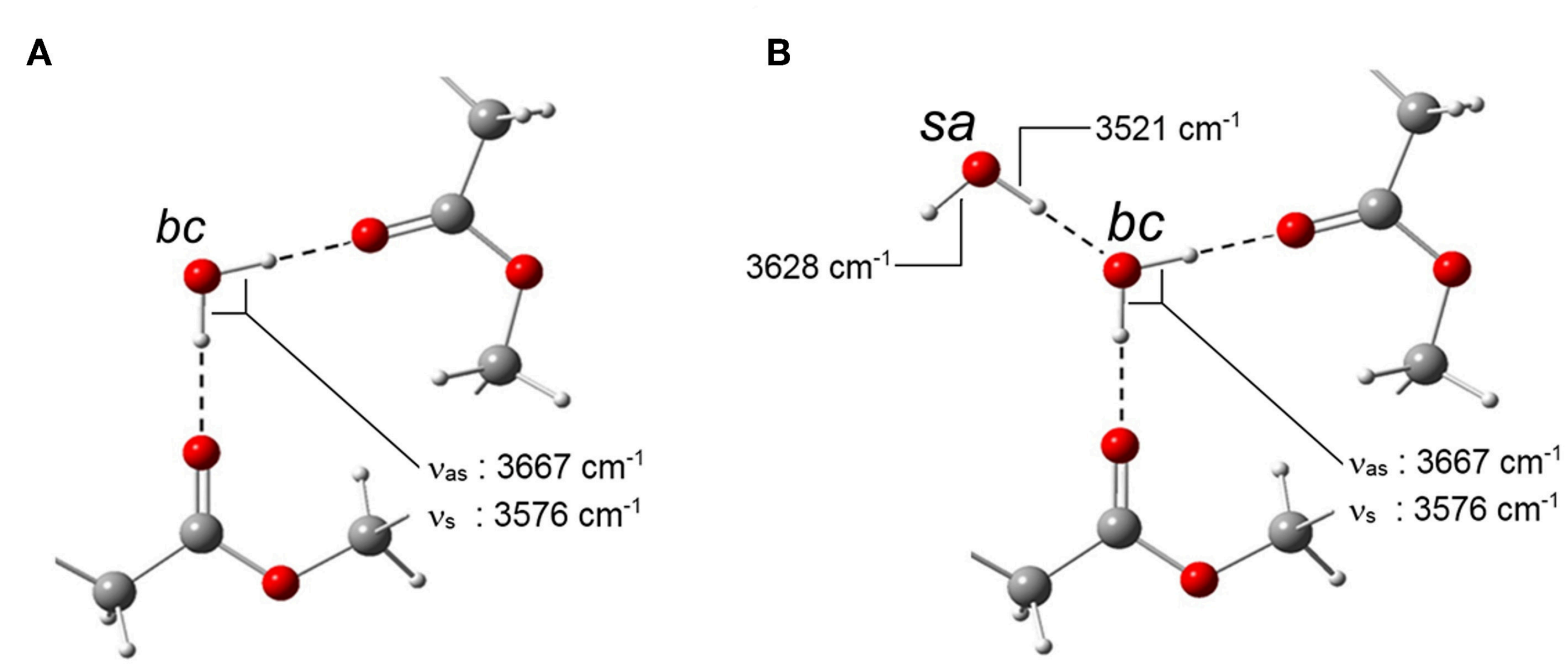
Schematic representation of the two water species identified in the H_2_O/PLLA system. **(A)** *bc* stands for *bound to carbonyls;* **(B)** *sa* stands for *self-associated*.

Of the two components belonging to the self-associated species, the one at 3,628 cm^−1^ originates (predominantly) from a “free” O–H bond, while that at 3,521 cm^−1^ is due to the O–H bond forming the self-interaction. In fact, the breadth of the latter band, around four times larger than the other three signals (see the forthcoming paragraph on LSCF analysis), is characteristic of water-to-water H-bonding, with the associated distribution of bond-lengths and geometries. The two water species identified spectroscopically with the signals they produce are schematically represented in Figure 8.

### LSCF Analysis

**Figure 9:**
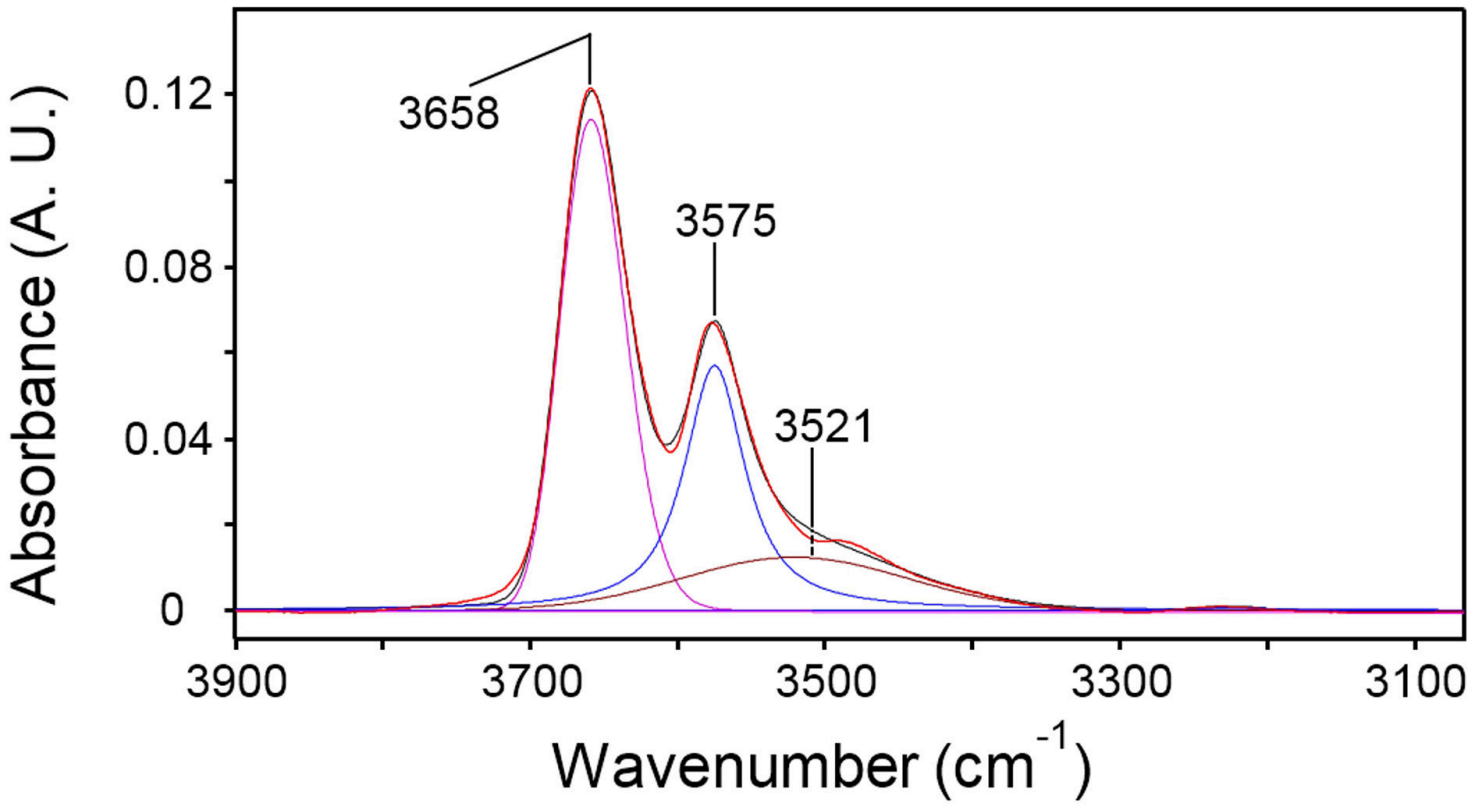
Curve fitting analysis of the spectrum representative of H_2_O sorbed in PLLA (*p/p*_0_ = 0.5). The figure displays the experimental profile (red trace), the best-fitting curve (black trace) and the resolved components.

The 2D-COS results were used as a benchmark to guide the LSCF analysis. Thus, a Gaussian component at 3,520 cm^−1^ was added to the model; the high-frequency peak was maintained as single component because no evidence of a fine structure was discernible. The regression of the spectrum representative of water sorbed at equilibrium in PLLA at *p/p*_0_ = 0.5 is reported in Figure 9.

The adopted model afforded a satisfactory and consistent simulation of all the experimental profiles collected at the different *p/p*_0_ values. The intensity of the resolved components can be converted into absolute concentration of the two water species provided that the values of the relative molar absorptivities, ε_*i*_, are known or can be estimated. In the present case, a method based on coupling the Beer-Lambert expression for the total concentration of sorbed water with the mass-balance relationship (Musto et al., 2014) was adopted. In brief,

(4) $$\begin{array}{l} C_{tot\;}=\;\frac{A_{bc}}{\varepsilon_{bc}L}+\frac{A_{sa}}{\varepsilon_{sa}L} \end{array}$$

(5) $$\begin{array}{l} C_{tot}=C_{bc}+C_{sa} \end{array}$$

(6) $$\begin{array}{l} \frac{A_{bc}}{C_{tot}}=\varepsilon_{bc}L-\frac{\varepsilon_{bc}}{\varepsilon_{sa}}\cdot \frac{A_{sa}}{C_{tot}} \end{array}$$

In Equations (4–6) *A* is the integrated absorbance, *C* the volumetric concentration and *L* the sample thickness. The subscripts *bc* and *sa* refer, respectively, to the H_2_O molecules bound to carbonyls and to those self-associated (see Figure 8); *tot* stands for total. The *C*_*tot*_ values were taken from the gravimetric measurements as a function of *p/p*_0_. The density of PLLA (1.240 g/cm^3^), assumed invariant with H_2_O sorption, was employed to convert gravimetric weight ratios into volumetric concentration values. The components at 3,575 and 3,521 cm^−1^ were selected as analytical peaks for the *bc* and the *sa* species, respectively, because of the unresolved, two-component structure of the 3,658 cm^−1^ peak.

**Figure 10:**
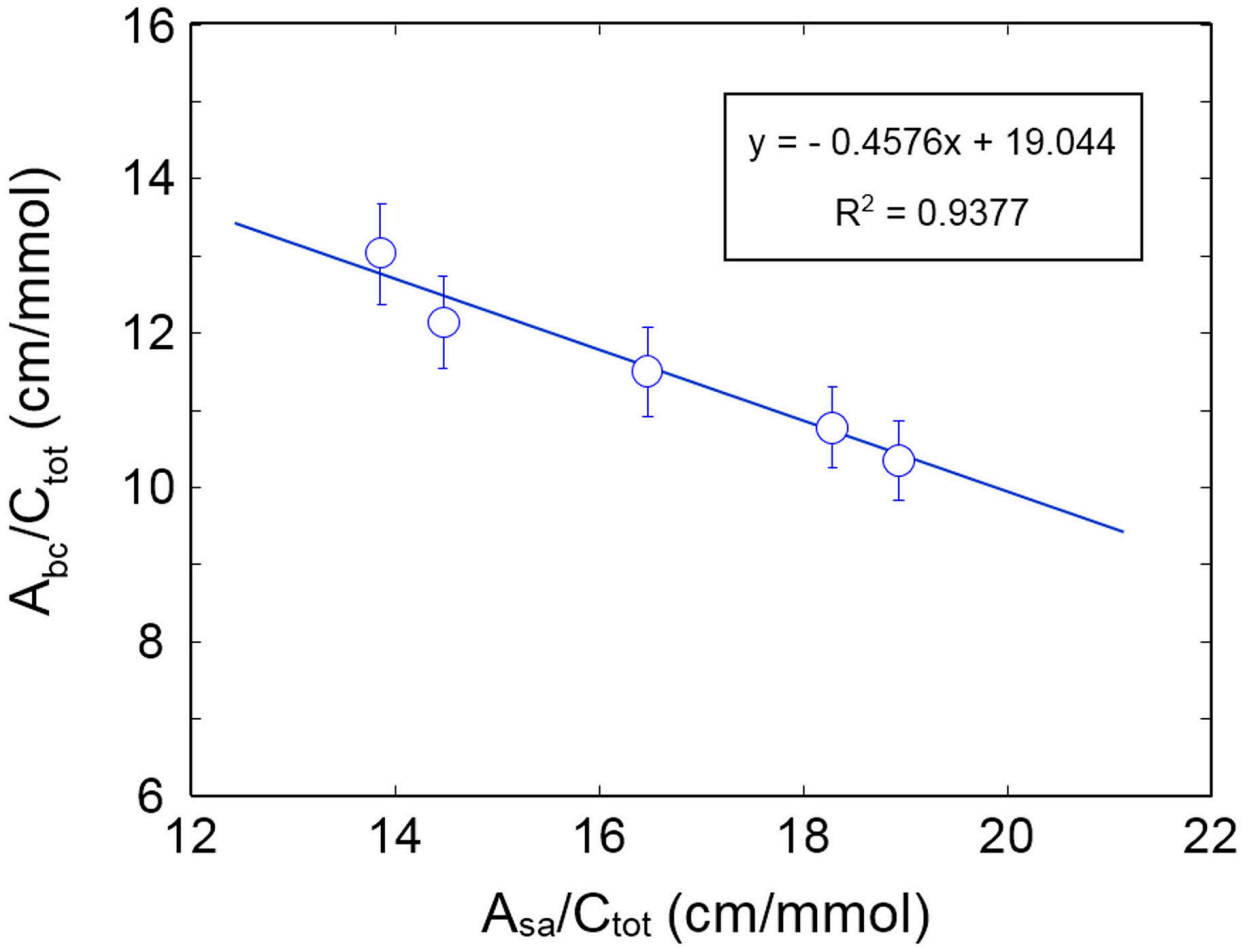
Plot of $\frac{A_{bc}}{C_{tot}}$ as a function of $\frac{A_{sa}}{C_{tot}}$.

The plot of $\frac{A_{bc}}{C_{tot}}\text{ vs}.\;\frac{A_{sa}}{C_{tot}}$ is displayed in Figure 10: the data exhibit the expected behavior. The absorptivity values calculated from the slope and the intercept of regression line are: ε_*bc*_ = 41.3 km/mol and ε_*sa*_ = 90.2 km/mol. These values compare favorably with those obtained for the system H_2_O/PCL (ε_*bc*_ = 72.2 km/mol and ε_*sa*_ = 98.2 km/mol) (Musto et al., 2014) and the system H_2_O/polyetherimide (ε_*bc*_ = 34.5 km/mol and ε_*sa*_ = 89.7 km/mol) (Musto et al., 2014; de Nicola et al., 2017).

**Figure 11:**
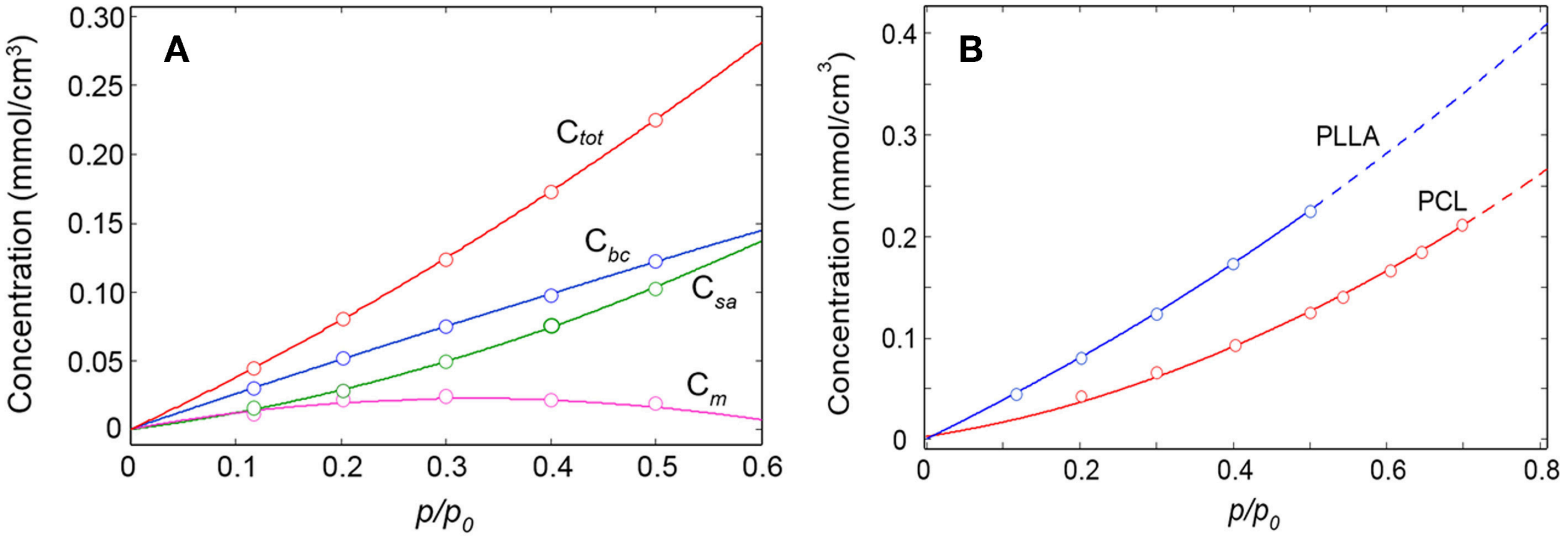
C_tot_, C_bc_, C_sa_, and C_m_ as a function of the relative pressure of water vapor **(A)**; comparison between the sorption isotherms of the H_2_O/PLLA and H_2_O/PCL systems **(B)**.

In Figure 11 are reported the absolute concentrations (mmol/cm^3^) of the two water species (calculated from the respective Lambert-Beer relationships) as a function of relative pressure of water vapor. In the same plot, for comparison, are also reported the total concentration of sorbed water (*C*_*tot*_) and the excess *C*_*bc*_ values, i.e., *C*_*m*_ = *C*_*bc*_−*C*_*sa*_.

In the light of the proposed structures of the H_2_O/PLLA molecular aggregates, *C*_*sa*_ corresponds to the concentration of H_2_O dimers and *C*_*m*_ to the concentration of isolated (monomeric) species in the system. The change of monomer concentration is modest, with a slight decreasing trend at high *p/p*_0_ values. In the whole pressure range the dimers represent the prevailing species. The *C*_*sa*_ curve displays an upward concavity not observed in the *C*_*bc*_ curve, which suggests an intersection of the two curves just above 0.6. At the intersection the isolated species are no longer present (*C*_*m*_ = 0) and all the H_2_O molecules bound to carbonyls are self-associated. If the *C*_*sa*_ offsets the *C*_*bc*_, aggregates of more than two water molecules are being formed in the system, which indicates the onset of the clustering process. In the present system this point lies above *p/p*_0_ = 0.5 and has not been reached (Musto et al., 2012; Scherillo et al., 2012a, 2014; Galizia et al., 2014).

It is informative to compare the sorption behavior of the H_2_O/PLLA and H_2_O/PCL systems (Musto et al., 2014) (see Figure 11B). Both substrates are aliphatic polyesters with a very close molecular structure (the only difference being in the aliphatic chain, comprising five CH_2_ groups for PCL and one –C(CH_3_)– unit for PLLA). PCL is semicrystalline with a crystallinity degree of 58% (DSC); the PLLA sample used in the present investigation is fully amorphous. The amount of water sorbed at equilibrium is significantly higher in PLLA than in PCL in the whole *p/p*_0_ range. This can be partially attributed to the higher interactive character of PLLA (higher density of C = O groups). The main effect is however related to the absence, in PLLA, of a crystalline phase impervious to the penetrant.

**Figure 12:**
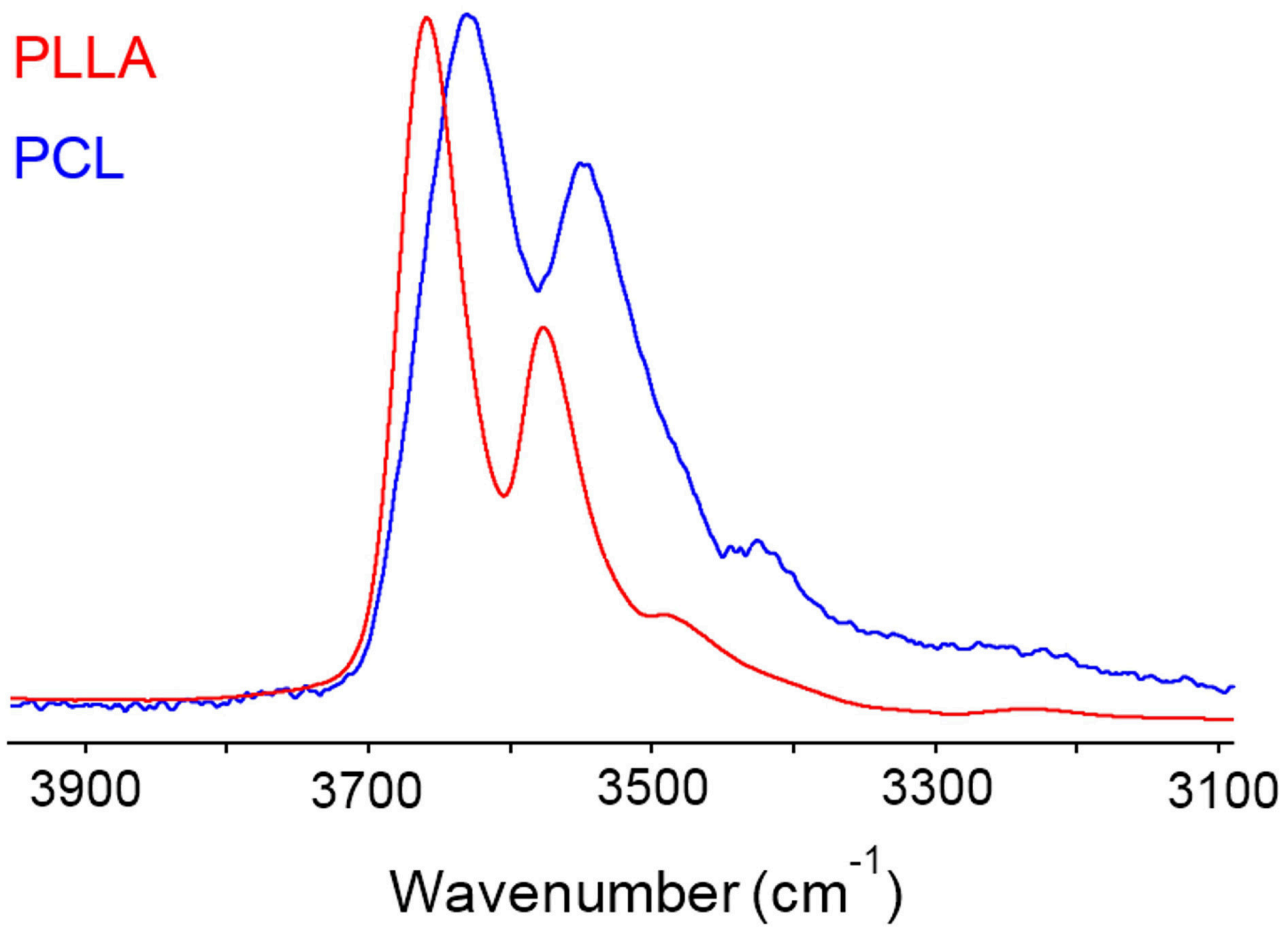
Spectra representative of water sorbed in PLLA (red trace) and in PCL (blue trace) in the ν(OH) region (3,900–3,100 cm^−1^).

In PCL the analysis of the two-species population indicated that up to 0.6 *p/p*_0_ values only dimers are formed, while at higher relative pressures of H_2_O vapor, clustering takes place (Musto et al., 2014). In PLLA, dimers are still the prevailing species, but monomeric water is present up to *p/p*_0_ = 0.5 and beyond. This result is again to be related to the higher number of proton acceptors (C = O) per unit volume in PLLA compared to PCL, which increases the probability for the penetrant of finding two close carbonyls in the right configuration for forming the aggregate represented in Figure 8A. The increased relevance of self-association in PCL is clearly reflected in the appearance of the penetrant spectrum (see Figure 12) where the broad component at lower frequency (self-associated O–H bond) is significantly larger than in PLLA.

## Conclusions

In the present contribution the sorption of water vapor in PLLA has been studied by FTIR spectroscopy. Data gathered at sorption equilibrium and during the diffusion process have been analyzed by different techniques, namely, difference spectroscopy, two-dimensional correlation spectroscopy and least-squares curve fitting, which provided complementary information. Two distinct molecular species were detected in the system: single H_2_O molecules bound to the PLLA carbonyls via a –C = O….H–O–H….O = C– stoichiometry and self-associated H_2_O molecules forming second- shell layers. No evidence was found of the presence of C = O….H–O–H species.

Coupling the spectroscopic data with gravimetric measurements, it was possible to evaluate the population of the two water species. It was found that, in the explored *p/p*_0_ range (0–0.5) dimers represent the prevailing species, but monomeric water remains well-detectable. In the present system the onset of the clustering process (i.e., when aggregates of more than two water molecules are formed) lies above *p/p*_0_ = 0.5 and has not been reached. The diffusion coefficient were measured as a function of water activity and is in good agreement with literature values. The diffusivity was found to increase with water concentration possibly due to a swelling effect.

## Author Contributions

MP: conceptualization, formal analysis, supervision, writing—review and editing. MP and PL: investigation. MP and PL: methodology.

### Conflict of Interest Statement

The authors declare that the research was conducted in the absence of any commercial or financial relationships that could be construed as a potential conflict of interest. The reviewer LV declared a shared affiliation, with no collaboration, with the authors, MP and PL, to the handling editor at the time of review. The reviewer GS declared a past co-authorship with one of the authors MP to the handling editor.
